# Mortality Rate Patterns for Hemorrhagic Fever with Renal Syndrome Caused by Puumala Virus

**DOI:** 10.3201/eid1610.100242

**Published:** 2010-10

**Authors:** Marika Hjertqvist, Sabra L. Klein, Clas Ahlm, Jonas Klingström

**Affiliations:** Author affiliations: Swedish Institute for Infectious Disease Control, Solna, Sweden (M. Hjertqvist, J. Klingström);; The Johns Hopkins Bloomberg School of Public Health, Baltimore, Maryland, USA (S.L. Klein);; Umeå University, Umeå, Sweden (C. Ahlm, J. Klingström);; Karolinska Institutet, Stockholm, Sweden (J. Klingström)

**Keywords:** Sex, age, case-fatality rate, hemorrhagic fever with renal syndrome, Puumala virus, hantavirus, nephropathia epidemica, viruses, Sweden, dispatch

## Abstract

To investigate nephropathia epidemica in Sweden during 1997–2007, we determined case-fatality rates for 5,282 patients with this disease. Overall, 0.4% died of acute nephropathia epidemica <3 months after diagnosis. Case-fatality rates increased with age. Only women showed an increased case-fatality rate during the first year after diagnosis.

Hantaviruses cause 2 severe emerging zoonotic diseases: hemorrhagic fever with renal syndrome (HFRS) in Eurasia and hantavirus cardiopulmonary syndrome (HCPS) in the Americas. These diseases have a case-fatality rate <40% depending on the specific hantavirus (*1**,**2*). Hantaviruses are primarily maintained in the environment by rodents, with humans serving as incidental hosts who are typically infected by inhalation of virus-contaminated rodent excreta (*1**,**2*). In parts of Europe, including Sweden, Puumala virus (PUUV) causes nephropathia epidemica (NE), a relatively mild form of HFRS with case-fatality rates of 0.1%–1% (*1**–**4*).

For many infectious diseases, frequency of infection is generally higher, and the clinical outcome often worse, in male patients (*5**–**8*). The reported male:female ratio for NE cases varies from 2 to 5:1 (*1**,**3*). Male-biased rates of infection have also been reported for other hantaviruses (*1**,**9*). However, in northern Sweden, seroprevalence does not differ by patient sex, which suggests the same number of persons of either sex might be infected with PUUV (*10*). Why males are overrepresented for diagnosed NE cases is not known.

We recently reported sex differences in cytokine responses during acute NE (*11*). Whether sexually dimorphic immune responses during hantavirus infection cause differences in the severity of disease between men and women has not been investigated. Furthermore, whether age is a risk factor in outcome of HFRS/HCPS is unknown. To investigate NE in Sweden during 1997–2007, we determined case-fatality rates for 5,282 patients with this disease.

## The Study

NE is a reportable disease according to the Swedish Communicable Disease Act. Clinicians and microbiologic laboratories report diagnosed cases to the Swedish Institute for Infectious Disease Control. These notifications are stored in a database. In this study, we included all 5,282 NE cases reported to the Swedish Institute for Infectious Disease Control and registered in the database during January 1, 1997–December 31, 2007. OpenEpi (www.openepi.com) was used to calculate the standardized mortality ratio (SMR). Because NE is an acute viral infection, and as a comparison for the numbers of deaths during the different phases of NE, we used the number of deaths during the second year after diagnosis in the studied population.

**Figure 1:**
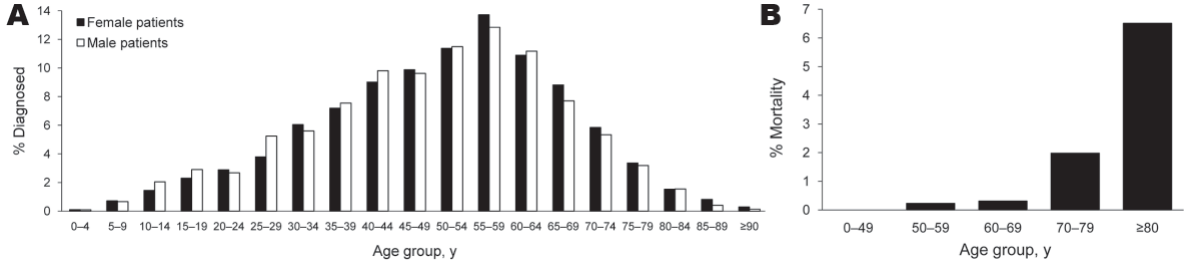
Incidence of acute nephropathia epidemica (NE) and case-fatality rates, Sweden, 1997–2007. A) Age distribution for male and female patients with acute NE. B) Age distribution of case-fatality rates for all patients with acute NE. The Swedish Death Register was used to identify all deceased persons with a diagnosis of NE. Numbers of deaths during different periods after diagnoses were 21 (13 male patients and 8 female patients) during the acute phase (<3 months after diagnoses), 7 (5 male patients and 2 female patients) after the acute phase (>3 months after diagnoses) but <1 year later, and 24 (19 male patients and 5 female patients) during the second year after diagnoses.

Incidence was highest in the group 55–59 years of age for men and women (Figure 1, panel A), and the mean ± SD age at diagnosis was 49.3 ± 16.7 years for men and 50.7 ± 16.5 years for women. The overall male:female ratio for NE cases was 1.52:1. Sex hormones can play major roles in susceptibility to infectious diseases (*5**,**7*). To test whether increased incidence of PUUV infection in men was dependent on sex hormones, we compared sex ratios in NE cases between children (persons <10 years of age and presumably prepubertal) and adults (>10 years of age). Sixty-two children were given a diagnosis of NE during 1997–2007. The male:female ratio for these children was 1.58:1, which was similar to the sex ratio for adult NE patients (1.52:1). This finding suggested that circulating sex hormones may not play a major role in the observed overrepresentation of NE in men.

Of persons with a diagnosis of NE, 21 (0.4%) patients died <3 months after diagnosis. The SMR was 3.5 (95% confidence interval [CI] 2.22–5.26) for all patients and 6.4 (95% CI 2.97–12.15) for female patients and 2.7 (95% CI 1.52–4.56) for male patients during the acute phase of NE.

No persons <50 years of age died during the acute phase (Figure 1, panel B). However, the case-fatality rate increased with age (Figure 1, panel B), and the case-fatality rate was 6.5% for patients >80 years of age, which showed age-dependent differences in mortality rates for NE. The mean ± SD age at time of death for patients with acute NE was 73.7 ± 10.4 years (range 50.9–88.5 years). Women died at a slightly, but not significantly, younger mean ± SD age (71.5 ± 12.4 years, range 50.9–85.7 years, n = 8) than men (75.1 ± 9.2 years, range 57.8–88.5 years, n = 13) (p = 0.347, by *t* test). Mean age at time of death for patients with NE was 10 years less than the expected life span for women and 3 years less than the expected life span for men (mean age at time of death in Sweden in 2003 was 78 years for men and 82 years for women; www.scb.se/statistik/_publikationer/BE0701_1986I03_BR_BE51ST0404.pdf).

**Figure 2:**
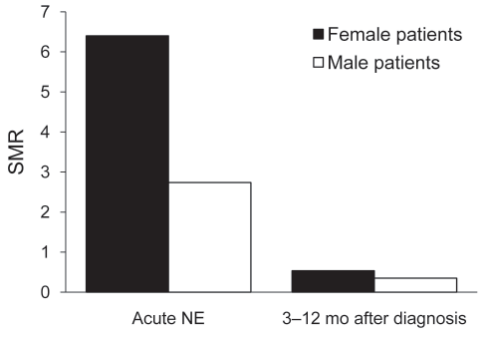
Overall standardized mortality ratios (SMRs) for male and female patients with a diagnosis of acute nephropathia epidemica (NE) and SMRs 3–12 mo after diagnosis, Sweden, 1997–2007.

Because no patient <50 years of age died of NE during the acute phase and the mean age at time of death was relatively high, we suggest that NE is rarely life threatening. To assess this possibility, we analyzed mortality rate patterns early after acute NE. The SMR decreased for the 9 months after acute NE in female (SMR 0.67, 95% CI 0.11–2.20) and male (SMR 0.36, 95% CI 0.13–0.79) patients (Figure 2).

We also analyzed SMRs for the first year (including the acute phase of NE [<3 months after diagnosis] and early phase [3–12 months] after NE diagnosis). For 52 persons who died <2 years after NE diagnosis, the case-fatality rate was only slightly higher in the first year (n = 28) than in the second year (n = 24) after diagnosis (SMR 1.17, 95% CI 0.79–1.66). A clear difference was observed between men and women. Deaths did not increase in men between the first (n = 18) and second (n = 19) years after diagnosis (SMR 0.95, 95% CI 0.58–1.49). However, we observed a 2× difference in deaths for women between the first (n = 10) and second (n = 5) years after diagnosis (SMR 2.0, 95% CI 1.02–3.57).

## Conclusions

We report an age-dependent case-fatality rate for the hantavirus disease NE; most deaths occurred in older persons. There is a report of patients <50 years of age dying of NE (*12*), but this finding is rare. Increased case-fatality rates in older persons have been described for other infectious diseases. For example, during a typical influenza season, 90% of deaths caused by influenza occur among persons >65 years of age (*13*). Whether this finding is unique to relatively mild infection with PUUV or is a conserved feature of all hantaviruses causing HFRS/HCPS is unknown.

We previously showed that there are sex differences in cytokine responses during acute NE (*11*), which suggested that there might be sex differences in severity of infection. Women showed a 2× difference in number of deaths between the first year and second year after NE diagnosis. However, men showed no difference in number of deaths between the first year and second year after diagnosis. These results suggest that there are sex differences in mortality rates after infection with PUUV. Whether NE is a more lethal disease in women than men or causes increased mortality rates in men up to 2 years after diagnoses is unknown.

The finding that the case-fatality rate for NE is associated with the age and sex of patients might have practical implications on healthcare issues. It may also indicate that age and sex should be considered predictive variables in clinical studies of hantavirus infections.
